# Supplementary material for: Mechanistic Investigation of the Formation of Nickel Nanocrystallites Embedded in Amorphous Silicon Nitride Nanocomposites
Source: Nanomaterials (Basel). 2022 May 11;12(10):1644. doi: 10.3390/nano12101644 (PMC9145008; doi:10.3390/nano12101644)
Supplement: Supplementary file 1 [file nanomaterials-12-01644-s001.zip › nanomaterials-1700100-supplementary.pdf]

nanomaterials

Supplementary Information

Mechanistic Investigation of the Formation of Nickel Nanocrystallites Embedded in Amorphous Silicon Nitride Nanocomposites

Norifumi Asakuma [1], Shotaro Tada [1], Erika Kawaguchi [1], Motoharu Terashima [1], Sawao Honda [1], Rafael Kenji Nishihora [2], Pierre Carles [2], Samuel Bernard [2] and Yuji Iwamoto [1,*]

[1] Department of Life Science and Applied Chemistry, Graduated School of Engineering, Nagoya Institute of Technology, Gokiso-cho, Showa-ku, Nagoya 466-8555, Japan; n.asakuma.633@stn.nitech.ac.jp (N.A.); tada.shotaro@nitech.ac.jp (S.T.); e.kawaguchi.310@stn.nitech.ac.jp (E.K.); m.terashima.311@stn.nitech.ac.jp (M.T.); honda@nitech.ac.jp (S.H.)

[2] CNRS, IRCER, UMR 7315, University of Limoges, F-87000 Limoges, France; rafael.nishihora@hotmail.com (R.K.N.); pierre.carles@unilim.fr (P.C.); samuel.bernard@unilim.fr (S.B.)

* Correspondence: iwamoto.yuji@nitech.ac.jp

Table S1. Intensity ratio of typical absorption bands for as-received PHPS and Ni-modifiedPHPS samples.

Sample	δN-H/νSi-N-Si	νSi-H/νSi-N-Si
**0.2NiPHPS**	0.213	0.425
**0.05NiPHPS**	0.290	0.583
**As-received PHPS**	0.346	0.671

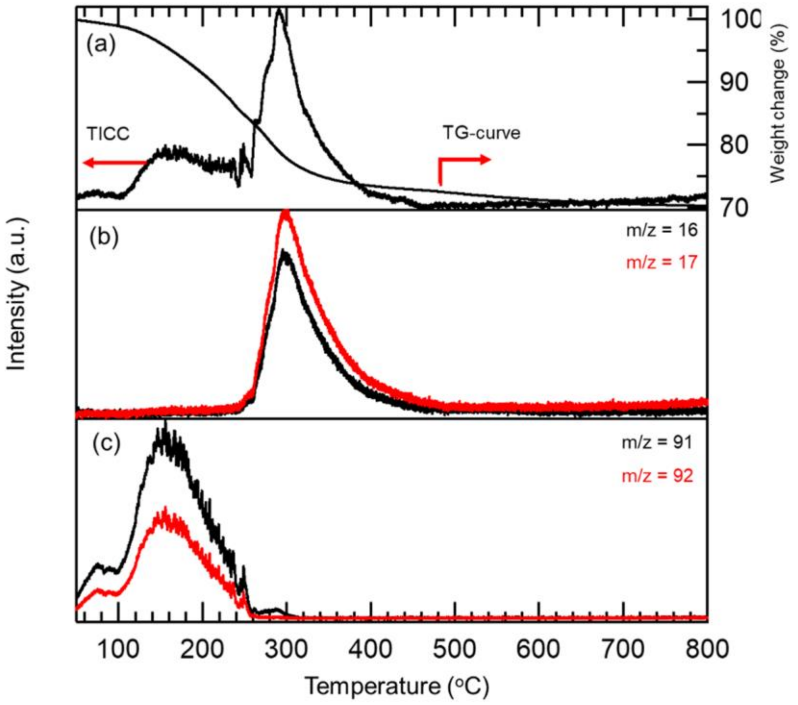

Figure S1. TG-MS analysis under flowing He of the as-received PHPS sample; (a) TG-curve coupled with the total ion current chromatogram, (b, c) the simultaneous monitoring of gaseousproducts formed in situ.

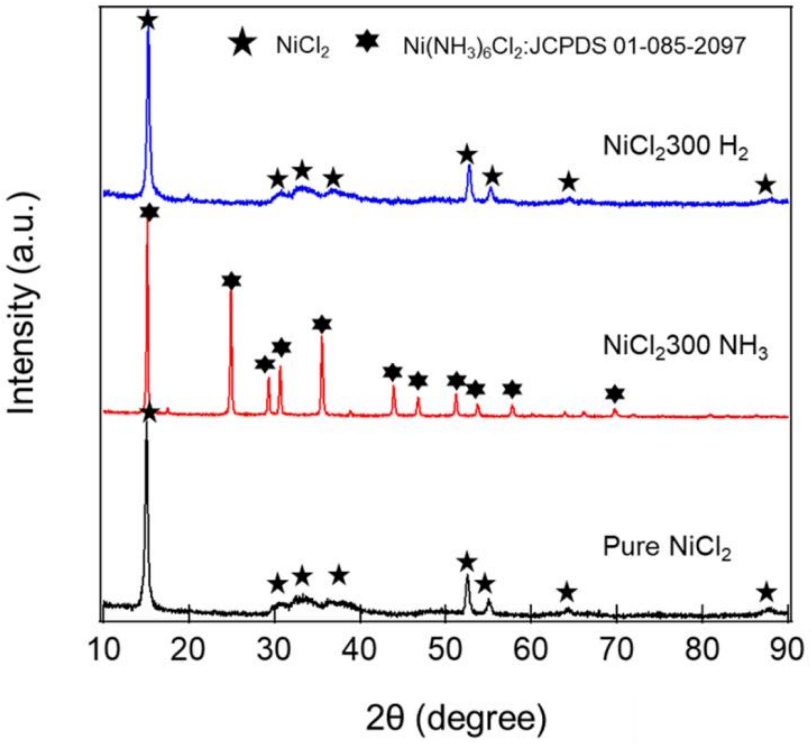

Figure S2. Powder X-ray diffraction patterns of pure $NiCl_2$ and pure $NiCl_2$ after pyrolysis at 300 °C under reductive gases (NH_3 and 10% H_2).

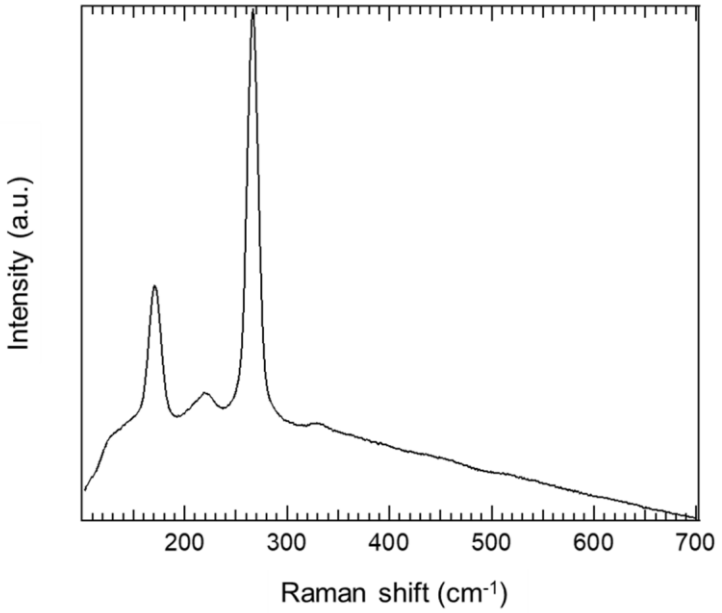

Figure S3. Raman spectrum of pure $NiCl_2$.

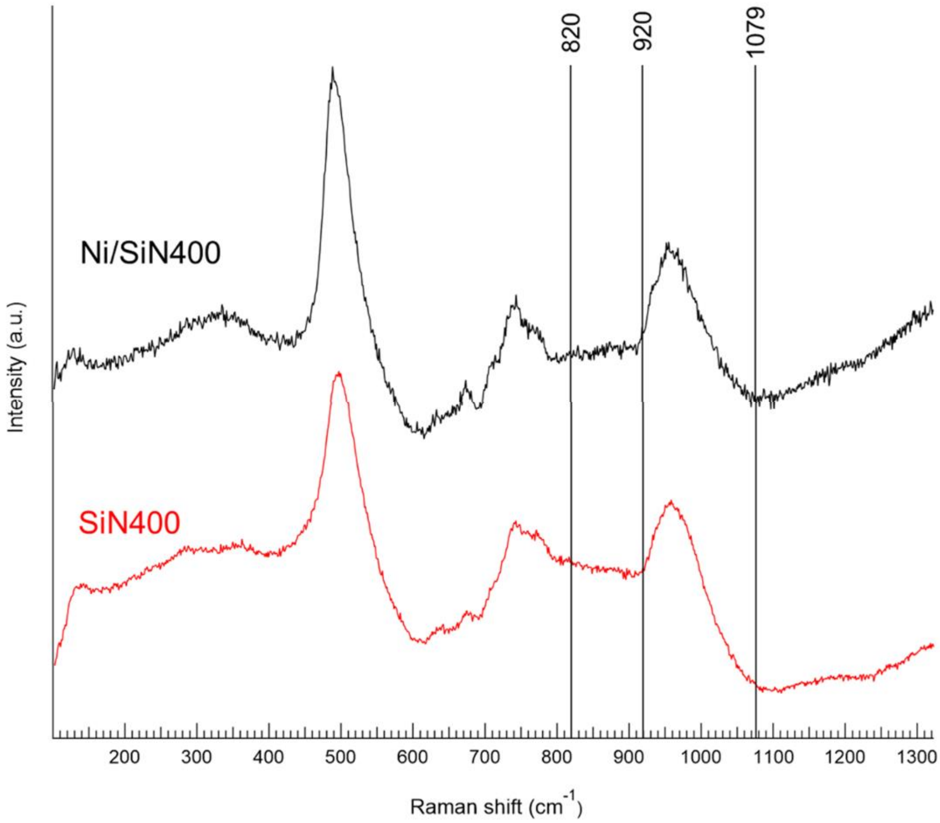

Figure S4. Raman spectra of Ni/SiN400 (black line) and SiN400 (red line) in wider Raman shift range.

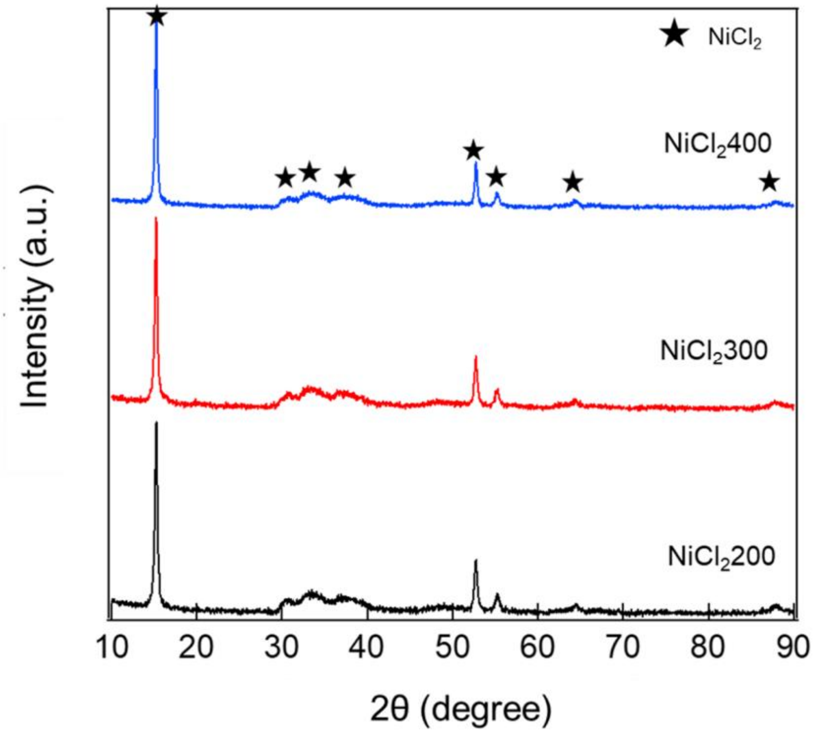

Figure S5. Powder X-ray diffraction patterns of NiCl2 after pyrolysis at 200, 300, 400 °C under the same manner for the pyrolysis of **0.05NiPHPS**.
